# Degraded and osteogenic properties of coated magnesium alloy AZ31; an experimental study

**DOI:** 10.1186/s13018-016-0362-5

**Published:** 2016-03-14

**Authors:** Jinpeng Zhuang, Yongbin Jing, Yaming Wang, Jinghuai Zhang, Huanxin Xie, Jinglong Yan

**Affiliations:** Department of Orthopedic Surgery, The 2nd Affiliated Hospital of Harbin Medical University, Harbin, Heilongjiang Province 150081 People’s Republic of China; Institute for Advanced Ceramics, Harbin Institute of Technology, Harbin, 150001 People’s Republic of China; Key Laboratory of Superlight Material and Surface Technology, Ministry of Education, Harbin Engineering University, Harbin, 150002 People’s Republic of China; No. 246, XuefuRoad, Nangang District, Harbin, Heilongjiang 150086 China

**Keywords:** Magnesium alloy, Degradation, Osteogenesis, Coating

## Abstract

**Background:**

Degraded and osteogenic property of coated magnesium alloy was evaluated for the fracture fixation in rabbits.

**Methods:**

Magnesium alloy AZ31 with a different coating thickness by microarc oxidation was used, and the bilateral radial fracture model was created by the bite bone clamp. Thirty-six New Zealand white rabbits in weight of 2.5~3.0 kg were randomly divided into A, B, and C groups at four time points and other 3 rabbits as the control group without magnesium alloy. Coated magnesium alloy AZ31 was implanted on the fracture and fixed with silk thread. Indexes such as general observation, histology, X-ray, hematology, and mechanical properties were observed and detected at 2nd, 4th, 8th, and 12th week after implantation.

**Results:**

Fracture in each rabbit was healed at 12th week after implantation. Among the three groups, the best results of general observation, histology, and X-ray appeared in A group without coating. However, A group showed the worst results from the perspective of mechanical properties about tensile strength and flexural strength, which failed to reach that of the natural bone at the 12th week. Comprehensive results displayed that C group with 20-μm coating was better than others in mechanical properties, while there is no difference between B and C groups in hematology.

**Conclusions:**

Degradation rate is inversely proportional to the coating thickness. And magnesium alloy with a 20-μm coating is more suitable for the fracture fixation.

## Background

Hard tissue material, as a kind of biological material, plays an important role in medicine. Stress shielding effect is one of the main defects during the internal fixation, which has a serious effect on bone remodeling process and affects the strength of new formed bone or even leads to fracture twice when internal fixation material was removed [1]. Magnesium is potentially attractive as a biomaterial because it is very lightweight, has a density similar to the cortical bone, has an elastic modulus close to the natural bone, is essential to human metabolism, is a cofactor for many enzymes, and stabilizes the structures of DNA and RNA [2]. Compared with other materials, magnesium-based implants may be degradable in vivo through simple corrosion and exhibit mechanical properties similar to the native bone, which can avoid the economic and psychological burdens of patients about a second surgery when it was taken out of fixation and residue, and had become one of the hot issues of domestic and foreign research.

Since 1808, when Davy discovered magnesium, people have tried to take it as an implant material for clinical service [3]. However, due to the rapid degradation of magnesium alloy in natural conditions, the application of it in clinical practice is limited for a long time [4]. In 1990, Payr first proposed that the magnesium alloy can be made into an internal fixation material such as a needle, wire, nail, and plate and used in fixed skeletal system [5]. In order for magnesium to be considered an acceptable biomaterial for tissue and bone replacement and regeneration, improvement of its corrosion resistance is needed. Thus, there have been methods developed in the art for the purpose of improving the corrosion resistance of magnesium. Known methods include element alloying and surface modification or coating [6].

The coated magnesium alloy is useful for tissue and bone repair and regeneration, such as but not limited to medical implant devices in orthopedic, craniofacial, dental, and cardiovascular surgeries [7]. Magnesium alloy AZ31 coated by microarc oxidation (MAO) has the advantages of good plasticity and mechanical strength and is a commonly wrought magnesium alloy [8]. Magnesium alloy, with its excellent mechanical properties, good biocompatibility, and biodegradable properties, has great potential as a new generation of hard tissue materials. With the different coating thickness of magnesium alloy used in rabbits in vivo, degraded and osteogenic properties of magnesium alloy were studied and the most suitable coating thickness as internal fixation was explored to provide valuable reference for clinical application.

## Methods

Thirty-nine New Zealand white rabbits (5 months) (provided by the Animal Experiment Center of Harbin Medical University) in weight of 2.5~3.0 kg either male or female were randomly divided into 13 groups. Except for one group without magnesium alloy after fracture which was considered as the control group (D group), other groups were divided into A, B, and C groups at four time points. According to the design of this study, magnesium alloy AZ31 (1 mm, composed of 2.5–3 % Al, 0.7–1.3 % Zn, >0.2 % Mn, and others Mg) was coated with a different thickness by MAO and cut into strips with 30 mm × 3 mm × 1 mm for implantation in vivo, while A group without coating was numbered 1–24, B group with 10-μm coating was numbered 25–48, and C group with 20-μm coating was numbered 49–72 (24 strips in each group). Coating thickness was measured by Minitest 600B (2 μm, EPK Company, Germany).

**Fig. 1:**
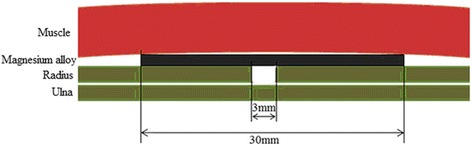
Implanted place of magnesium alloy AZ31 in vivo

The bilateral radial fracture model was created by the bite bone clamp and about 3-mm wound was made, removing the periosteum and substance. Before implantation, rabbits were anesthetized at the ear vein by slow injection of 10 % chloralhydrate solution (2 ml/kg). Coated magnesium alloy AZ31 was implanted on the wound and fixed with silk thread which is shown in Fig. 1. Then, the affected limbs were fixed with bandage.

Every rabbit had two wounds, also known as experimental site, in the left and right front legs, respectively. Second, 4th, 8th, and 12th week after implantation were set as observed time. Aerodermectasia, flare, excreta, and movement of the rabbits at four time points were checked and then rabbits were killed to observe and photograph the repaired situation of the wound. Moreover, magnesium alloy AZ31 was taken out to check the coating surface and test the maximum tensile strength and flexural strength (15-mm span and 1-mm/min acceleration) in each group. Blood from the heart was taken and sent to the laboratory for the detection of Mg^2+^. X-ray (Siemens 1350, Germany) was used to photograph and observe the healing conditions of the fracture. In addition, decalcified bone slices and routine heart and kidney tissue slices were made and stained with hematoxylin-eosin (HE), while preliminary observation of sections was done under an optical microscope (Olympus, Japan).

The software SAS 9.1.3 was used to analyze data. All data were expressed by mean ± SEM. Analysis of variance (ANOVA) and factorial design ANOVA were used and *P* values less than 0.05 were considered significantly.

This study was approved by the ethics committee of the 2nd Affiliated Hospital of Harbin Medical University and was conducted in accordance with the provisions of the Declaration of Helsinki, Good Clinical Practice guidelines, and local laws and regulations.

## Results

Rabbits began to wake up 1 h after the implantation and can carry out normal activities. One day later, two front legs can be slightly stressed. The implanted magnesium alloy AZ31 was coated with a thin layer of fibrous tissue at four time points and new bone tissue appeared from 4th week, while A group was with the best healing situation and B group was better than C group.

**Fig. 2:**
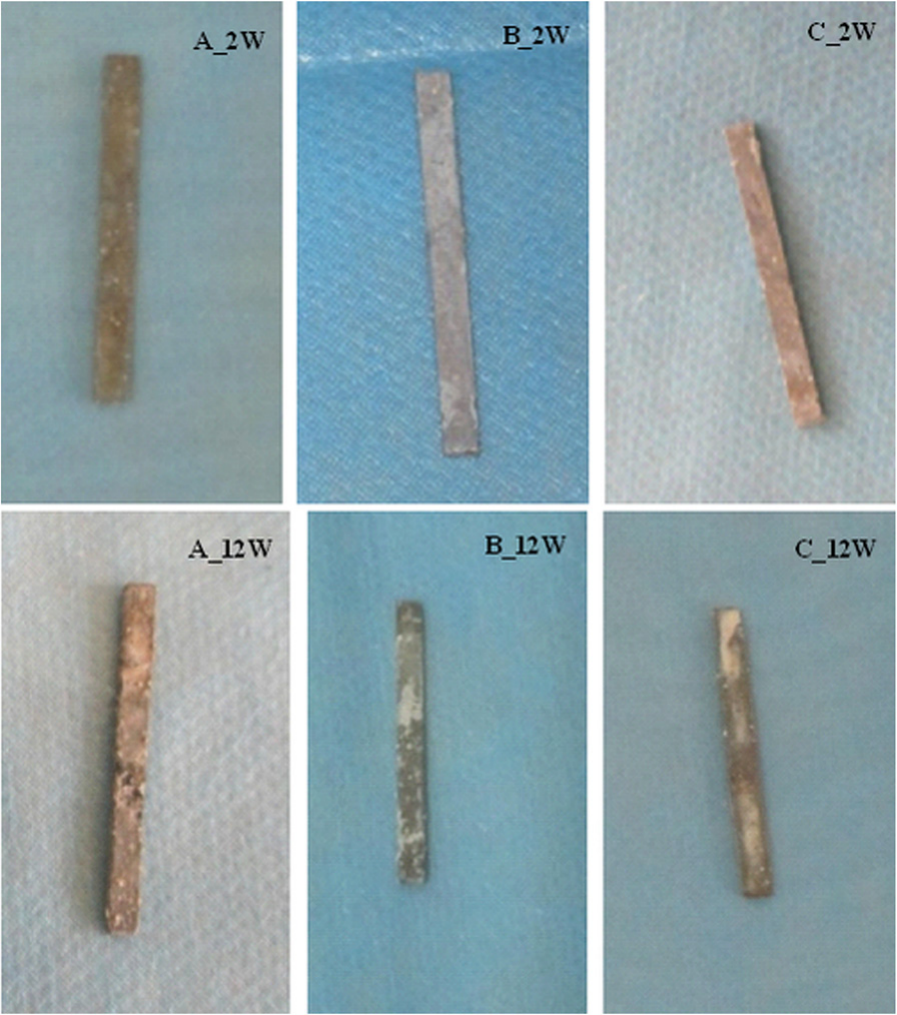
The changes of magnesium alloy AZ31 from 2nd week to 12th week. Group A means magnesium alloy AZ31 without coating. Group B means magnesium alloy AZ31 with 10-μm coating. Group C means magnesium alloy AZ31 with 20-μm coating

The changes of magnesium alloy AZ31 are shown in Fig. 2. At the 12th week, the magnesium alloy appeared with severe corrosion and became thin but not broken in A group, the magnesium alloy matrix was corroded seriously in B group with a coverage less than 50 %, and a large area of coating disappeared and the magnesium alloy matrix was only lightly corroded with a coverage of more than 50 %.

**Fig. 3:**
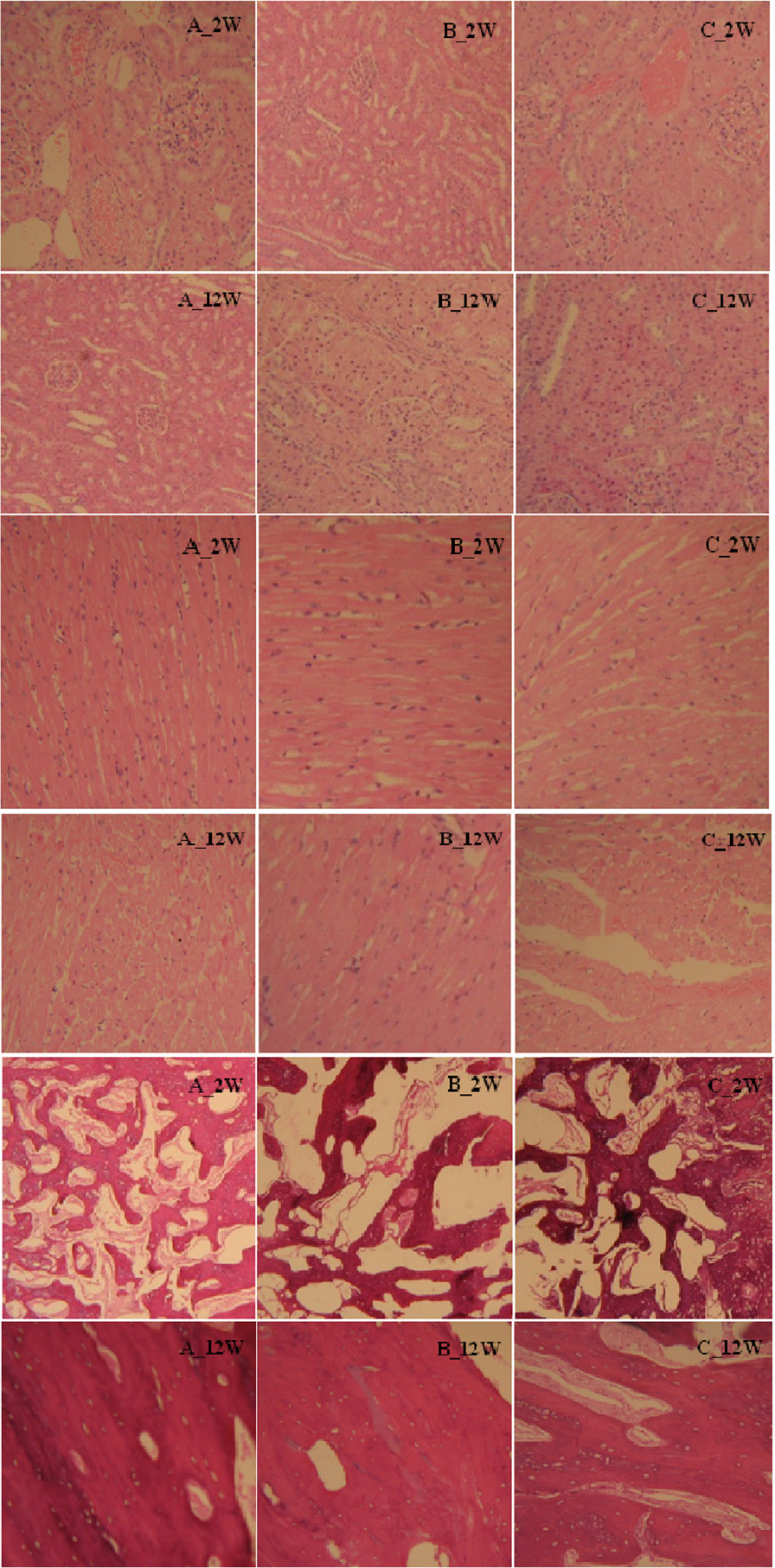
HE staining results of the kidney tissue, heart tissue, and decalcified bone slices. Group A means magnesium alloy AZ31 without coating. Group B means magnesium alloy AZ31 with 10-μm coating. Group C means magnesium alloy AZ31 with 20-μm coating

**Fig. 4:**
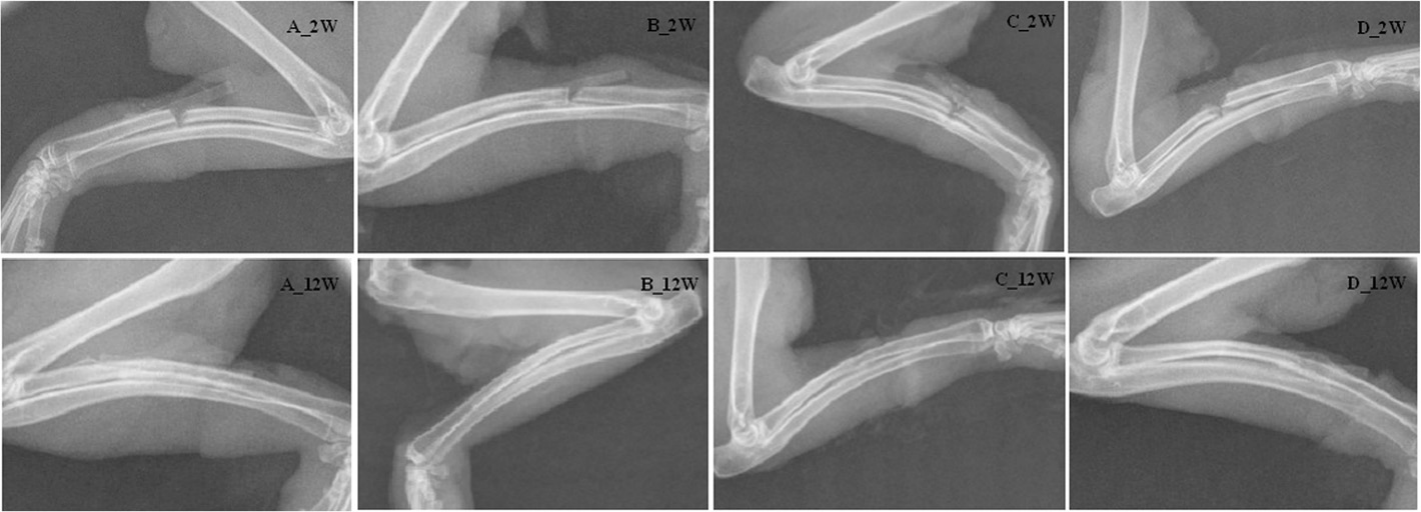
Comparison of the bone tissue at 2nd week and 12th week. Group A means magnesium alloy AZ31 without coating. Group B means magnesium alloy AZ31 with 10-μm coating. Group C means magnesium alloy AZ31 with 20-μm coating. Group D means the control group

The results of HE staining show that the shape and volume of the glomerulus and myocardial cell appeared normal at four time points (Fig. 3). Bone histological examination results show the maturity of bone spicules in A group was high. Although bone spicules were not as well as A and B groups, the direction of bone spicules was all the same. In addition, the results of X-ray were shown in Fig. 4, in which the fractures were completely healed at the 12th week and magnesium alloy was kept well. Hematology test of Mg^2+^ was checked and the results were displayed in Table 1. Factorial design ANOVA was done. The results were shown in Table 2, in which there was no statistical significance between B and C groups (*P* = 0.1189) (Table 3).

**Table 1:** Hematology test of magnesium in four groups (mmol/L, mean ± SEM)

Group	2nd week	4th week	8th week	12th week	*P* value
A	1.68 ± 0.11	1.51 ± 0.18	1.75 ± 0.12	1.64 ± 0.20	<0.0001*
B	1.36 ± 0.22	1.40 ± 0.06	1.42 ± 0.24	1.68 ± 0.39	0.0171**
C	1.31 ± 0.08	1.50 ± 0.23	1.31 ± 0.11	1.29 ± 0.18	0.0003***
Control	1.08 ± 0.09	1.02 ± 0.09	0.97 ± 0.05	1.06 ± 0.09	

*Compared A group with the control group

**Compared B group with A group

***Compared C group with A group

**Table 2:** Factorial design analysis of variance by hematology test results

Variation	Freedom	SS	MS	*F*	*P*
Total variation	47	3.7401			
Group	3	2.3743	0.7914	25.98	<0.0001
Time	3	0.0316	0.0105	0.35	0.7923
Group × time	9	0.3593	0.0399	1.31	0.2701
Error	32	0.9748	0.0305

**Table 3:** Comparison between two groups of hematology test results

Group 1	Group 2	*t*	*P*
A	B	2.5145	0.0171
A	C	4.1167	0.0003
A	D	8.5727	<0.0001
B	C	1.6023	0.1189
B	D	6.0582	<0.0001
C	D	4.4559	<0.0001

α = 0.05/6 = 0.0083

*Group A* means magnesium alloy AZ31 without coating. *Group B* means magnesium alloy AZ31 with 10-μm coating. *Group C* means magnesium alloy AZ31 with 20-μm coating. *Group D* means the control group

Mechanical properties were tested after magnesium alloy AZ31 was taken out from the rabbits. The results of tensile strength and flexural strength were displayed in Tables 4 and 5, respectively. The decrease of tensile strength released the rule of firstly slow and then fast in A, B, and C groups. Furthermore, there was statistical significance between any two groups at four time points (*P* < 0.05) (Table 6). Flexural strength showed the same rule with tensile strength.

**Table 4:** Results of tensile strength in A, B, and C groups (MPa, mean ± SEM)

Group	2nd week	4th week	8th week	12th week
A	177.08 ± 2.25	161.56 ± 1.54	135.16 ± 1.66	112.92 ± 1.00
B	184.99 ± 0.61	171.93 ± 7.97	154.06 ± 1.85	132.25 ± 7.42
C	206.32 ± 8.50	195.28 ± 6.16	174.38 ± 1.98	153.40 ± 1.68

*Group A* means magnesium alloy AZ31 without coating. *Group B* means magnesium alloy AZ31 with 10-μm coating. *Group C* means magnesium alloy AZ31 with 20-μm coating

**Table 5:** Results of flexural strength in A, B, and C groups (MPa, mean ± SEM)

Group	2nd week	4th week	8th week	12th week
A	266.45 ± 2.57	241.88 ± 0.86	202.72 ± 3.14	148.34 ± 2.66
B	307.45 ± 1.78	281.78 ± 5.96	244.30 ± 8.00	184.28 ± 3.94
C	319.57 ± 8.85	296.25 ± 2.74	260.66 ± 2.69	217.43 ± 2.21

*Group A* means magnesium alloy AZ31 without coating. *Group B* means magnesium alloy AZ31 with 10-μm coating. *Group C* means magnesium alloy AZ31 with 20-μm coating

**Table 6:** Significant analysis of in A, B, and C groups

Time	Group 1	Group 2	*P* (tensile strength)	*P* (flexural strength)
2nd week				
	A	B	0.0445	<0.0001
	A	C	<0.0001	<0.0001
	B	C	<0.0001	0.0191
4th week				
	A	B	0.0104	<0.0001
	A	C	<0.0001	<0.0001
	B	C	<0.0001	0.0072
8th week				
	A	B	<0.0001	<0.0001
	A	C	<0.0001	<0.0001
	B	C	<0.0001	0.0033
12th week				
	A	B	<0.0001	<0.0001
	A	C	<0.0001	<0.0001
	B	C	<0.0001	<0.0001

*Group A* means magnesium alloy AZ31 without coating. *Group B* means magnesium alloy AZ31 with 10-μm coating. *Group C* means magnesium alloy AZ31 with 20-μm coating

## Discussion

Although general observation, X-ray, and bone tissue section of A group without coating showed the best results, the flexural strength and tensile strength were the lowest at four time points and cannot reach the requirements from the view of the mechanical properties of magnesium alloy. In general, mechanical properties of C group with 20-μm coating were better than that of B group with 10-μm coating, excepting for no difference of hematology; others such as histology and X-ray were all in turn. However, higher requirements of internal fixation about mechanical properties are needed clinically in the fracture healing process.

The internal fixation is an important part of medical treatment, which needs to be constantly improved. At present, the metal and polymer are the two main types of internal fixation material in the clinic which have advantages but also a lot of problems. The ideal internal fixation material should have the following characteristics [9]: (1) good biocompatibility, (2) no side effects such as carcinogenic, mutagenic, and toxic, (3) sufficient mechanical strength without stress shielding effect, (4) biodegradable and absorbable property to avoid economic and spiritual burden caused by the second surgery, (5) biological activity to promote fracture healing. Thus, finding a more suitable internal fixation material has become one of the hotspot problems.

Magnesium alloy, as a kind of biodegradable material, is degradable in vivo through simple corrosion and exhibits mechanical properties similar to the native bone. The elastic modulus (35–45 GPa) is very close to that of the human bone and can effectively avoid stress shielding effect. At the same time, the degradation of magnesium alloy can form an alkaline environment neutralizing acid environment caused by inflammatory reaction, which can stimulate the secretion of bone morphogenetic proteins promoting bone formation [10]. Wrought magnesium alloy does not only have high strength but also have good ductility and can satisfy the need of medical implants about different shapes. After the implantation in vivo, the magnesium alloy will release an amount of hydrogen, which can be controlled by adjusting the degradation rate [11]. A large amount of hydrogen can be discharged from the body through the exhaust needle and a small amount of gas can be removed out of the body through the blood circulation and metabolism. In vivo, Mg^+^ has the antagonism of vascular contraction and blood coagulation caused by calcium ion. Moreover, the magnesium ion also can dilate blood vessels, reduce the formation of thrombus, and control the disease such as arrhythmia effectively [5]. In addition, the magnesium alloy can be implanted into the body several times. Compared with the titanium alloy used in clinic at the present, the magnesium alloy has a significant effect on the formation of a new bone [12].

If the defect size of mid-radius was more than or equal to 1.4 cm, the defect will not repair itself which had been reported by Prof. Zhao [13]. Due to the congenital osseous fusion of the ulna and radius in the two front legs of rabbit, the ulna can play a role of support after fracture and external fixation treatment is not required. Meanwhile, other factors such as shear stress on the implanted materials can be avoided [9]. In this study, the bilateral radial fracture model was created by the bite bone clamp and the wound was 3 mm, maintaining the consistency of fracture model and avoiding the influence of other factors. In addition, surface modification by MAO which is a very promising technology of implant has been successfully applied to the surface of titanium implants [14]. MAO has the characteristics of firmness, compactness, high toughness, and resistance to wear, corrosion, and high temperature. What is more, the coating thickness can be adjusted according to the voltage and current, and Ca and P elements can be deposited on the surface according to the different electrolyte which is helpful to the adsorption of osteoblasts [11]. Coating by MAO can effectively reduce the degradation rate of magnesium alloy; the produced Mg(OH)_2_ covered on the surface can also inhibit the degradation of the magnesium alloy.

Regenerated bone was always found around the implanted magnesium alloy, which is derived from neutralization of OH^−^ produced by the degradation of magnesium alloy and the acidic substances produced by inflammatory reaction stimulating the secretion of bone morphogenetic proteins. At the same time, Mg^2+^ is one of the components of the bone and can promote the deposition of calcium salt, which can also accelerate the formation of the bone and promote fracture healing [5]. So, A group without coating released the best result of healing situation. Magnesium alloy, after all, belongs to substance in vitro and will cause foreign body reaction after implantation in vivo but which is relatively minor and will not cause damage to the body. Local emphysema produces local swelling and pain symptoms without other adverse effects at present. Through the observation about tissue pathological section of the heart and kidney, no obvious abnormality was found. Therefore, the implantation of magnesium alloy has no negative impact on the circulatory system and urinary system of the rabbits.

The ultimate goal of bone healing is to restore the integrity and continuity of the bone. This study tries to look for a more suitable coating thickness of magnesium alloy as a fixation for fracture healing, at the same time, also with characteristics of accelerating the healing of fracture, even with mechanical strength similar to the natural bone. Destructive mechanics test was used in this study to show the degradation of magnesium alloy in the body from two aspects like the maximum flexural strength and the maximum tensile strength [15]. The results showed that only the flexural strength and tensile strength of C group with 20-μm coating reached or even more than the mechanical strength of the human femur at 12th week after fracture, while A and B groups did not reach that.

## Conclusions

Degradation rate is inversely proportional to the coating thickness. And the comprehensive results of all showed that magnesium alloy AZ31 with 20-μm coating is more suitable for internal fixation.
